# Supplementary material for: The Small RNA Universe of Capitella teleta
Source: Front Mol Biosci. 2022 Feb 25;9:802814. doi: 10.3389/fmolb.2022.802814 (PMC8915122; doi:10.3389/fmolb.2022.802814)
Supplement: Supplementary file 1 [file DataSheet1.ZIP › Supplement/known/CAPTEscaffold_910_30367.pdf]

Provisional ID	:	CAPTEscaffold_910_30367
Score total	:	775844.8
Score for star read(s)	:	3.9
Score for read counts	:	775834.2
Score for mfe	:	2.1
Score for randfold	:	1.6
Score for cons. seed	:	3
Total read count	:	1521777
Mature read count	:	1505732
Loop read count	:	516
Star read count	:	15529

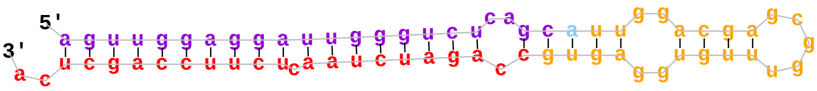

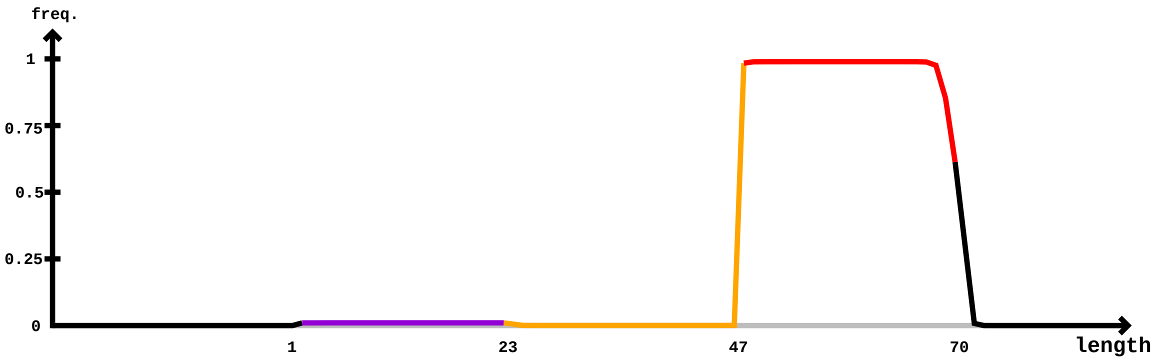

Star Mature

```
5'- cuugauguucugccaggugcgaugaguuggaggauugggucucagcauuggacgagcgguuuguggagugccagaucuaacucuuccagcucaucccacucuggaccucc -3'   obs
    cuugauguucugccaggugcgaugaguuggaggauugggucucagcauuggacgagcgguuuguggagugccagaucuaacucuuccagcucaucccacucuggaccucc        exp
    cuugauguucugccaggugcgaugaguuggaggauugggucucagcauuggacgagcgguuuguggagugccagaucuaacucuuccagcucaucccacucuggaccucc        known
    ............(((((((.((((((((((((((((((((((..(((((..((((.....))))..))))).)))))))).)))))))))))))).))).))))......        reads   mm   sample
    .............caggugcgaugaguuggaggauugggucuca..................................................................        1       0    seq
    ......................ugaguuggaggauugggucucagcauuggac.........................................................        1       0    seq
    ........................aguuggaggauugggucu....................................................................        1       0    seq
    ........................aguuggaggauugggucuc...................................................................        43      0    seq
    ........................aguuggaggauugggucuca..................................................................        19      0    seq
    ........................aguuggaggauugggucucag.................................................................        160     0    seq
    ........................Gguuggaggauugggucucag.................................................................        1       1    seq
    ........................aguuggaggauuAggucucag.................................................................        1       1    seq
    ........................aguuAgaggauugggucucag.................................................................        2       1    seq
    ........................aguuggGggauugggucucagc................................................................        4       1    seq
    ........................aguugCaggauugggucucagc................................................................        1       1    seq
    ........................aguuggagUauugggucucagc................................................................        2       1    seq
    ........................aguuUgaggauugggucucagc................................................................        3       1    seq
    ........................Nguuggaggauugggucucagc................................................................        2       1    seq
    ........................aguuggaggaAugggucucagc................................................................        3       1    seq
    ........................aguuggaggauugggucucGgc................................................................        3       1    seq
    ........................aguCggaggauugggucucagc................................................................        2       1    seq
    ........................aguuggaggauugggucucaUc................................................................        1       1    seq
    ........................aguuggaggauugAgucucagc................................................................        3       1    seq
    ........................aguuCgaggauugggucucagc................................................................        1       1    seq
    ........................aguuggaggauugggucuAagc................................................................        1       1    seq
    ........................Uguuggaggauugggucucagc................................................................        1       1    seq
    ........................aguuggaggauugggucucagU................................................................        1       1    seq
    ........................aguuggaggauCgggucucagc................................................................        2       1    seq
    ........................aguuggaggGuugggucucagc................................................................        2       1    seq
    ........................agAuggaggauugggucucagc................................................................        6       1    seq
    ........................aguuggaggUuugggucucagc................................................................        1       1    seq
    ........................aguuggaggauugggucGcagc................................................................        2       1    seq
    ........................Gguuggaggauugggucucagc................................................................        66      1    seq
    ........................aguuggaggauugggCcucagc................................................................        2       1    seq
    ........................aguuggaggauugggucucagA................................................................        6       1    seq
    ........................aguuggaggauuggCucucagc................................................................        2       1    seq
```

Star   Mature

```
cuugauguucugccaggugcgaugaguuggaggauugggucucagcauuggacgagcgguuuguggagugccagaucuaacucuuccagcucaucccacucuggaccucc
........................aguuggaggauuAggucucagc................................................................  29     1  seq
........................aguuggaggauugggucucUgc................................................................  1      1  seq
........................aguuggaggauugggucAcagc................................................................  2      1  seq
........................aguugAaggauugggucucagc................................................................  1      1  seq
........................aguuggaAgauugggucucagc................................................................  10     1  seq
........................aguuggaggauuUggucucagc................................................................  4      1  seq
........................aguuAgaggauugggucucagc................................................................  42     1  seq
........................aguAggaggauugggucucagc................................................................  3      1  seq
........................aguugUaggauugggucucagc................................................................  2      1  seq
........................agGuggaggauugggucucagc................................................................  1      1  seq
........................aguuggaggauugggucuUagc................................................................  7      1  seq
........................aguuggaggauugggucucagG................................................................  2      1  seq
........................aguuggaggaCugggucucagc................................................................  2      1  seq
........................aguuggaggauugggAcucagc................................................................  3      1  seq
........................aguuggaggauuggguAucagc................................................................  1      1  seq
........................aguuggaggauugggucucagc................................................................  13116  0  seq
........................aguuggaggauAgggucucagc................................................................  3      1  seq
........................aguuggaggaGugggucucagc................................................................  3      1  seq
........................aguuggaggauugggucucagcU...............................................................  31     1  seq
........................aguuggaggauugggucucagcG...............................................................  1      1  seq
........................aguuggaggauugggucucUgca...............................................................  1      1  seq
........................agAuggaggauugggucucagca...............................................................  1      1  seq
........................aguuggaggauugggAcucagca...............................................................  1      1  seq
........................aAuuggaggauugggucucagca...............................................................  1      1  seq
........................Nguuggaggauugggucucagca...............................................................  1      1  seq
........................aguuUgaggauugggucucagca...............................................................  1      1  seq
........................aguuggaggauuAggucucagca...............................................................  6      1  seq
........................aguuggaggauugggucuUagca...............................................................  2      1  seq
........................aguuggaggauugggucucagca...............................................................  1553   0  seq
........................aguuggaggauugggucAcagca...............................................................  1      1  seq
........................aguuggaggUuugggucucagca...............................................................  1      1  seq
........................aguuAgaggauugggucucagca...............................................................  12     1  seq
........................aguuggaggauuggUucucagca...............................................................  1      1  seq
........................Gguuggaggauugggucucagca...............................................................  11     1  seq
........................aguuggaUgauugggucucagca...............................................................  1      1  seq
........................aguugUaggauugggucucagca...............................................................  1      1  seq
........................aguuggaggauCgggucucagca...............................................................  1      1  seq
........................aguuggaggauugggucucagcau..............................................................  5      0  seq
........................aguuAgaggauugggucucagcau..............................................................  1      1  seq
........................aguuggaggauugggucucagcauu.............................................................  2      0  seq
........................aguuggaggauugggucucagcauA.............................................................  42     1  seq
........................aguuggaggauugggucucagcauC.............................................................  1      1  seq
.........................guuggaggauugggucucagc................................................................  1      0  seq
.........................Auuggaggauugggucucagc................................................................  1      1  seq
..........................uuggaggauugggucucagc................................................................  1      0  seq
..........................uuggaggauugggucuUagc................................................................  93     1  seq
..........................uuggaggauugggucucagcau..............................................................  77     0  seq
..........................uuggaggauugggucucagcauuggacgagc.....................................................  3      0  seq
...........................uggaggauugggucucagc................................................................  1      0  seq
...........................uggaggauugggucucagcau..............................................................  2      0  seq
...........................uggaggauugggucucagcauu.............................................................  96     0  seq
...........................uggaggauugggucucagcaAu.............................................................  1      1  seq
...........................uggaggauuCggucucagcauu.............................................................  1      1  seq
...........................uggaggauugggucucagcauuggac.........................................................  1      0  seq
.........................................ucagcauuggacgagcgguuuguggagugc.......................................  2      0  seq
...............................................uuggacgagcgguuuguA.............................................  1      1  seq
...............................................uuggacgagcgguuugugga...........................................  2      0  seq
...............................................uuAgacgagcgguuuguggag..........................................  1      1  seq
...............................................uuggacgagcgguuuguggag..........................................  30     0  seq
...............................................uuggacgagcgguuuguggagu.........................................  23     0  seq
...............................................uuAgacgagcgguuuguggagug........................................  3      1  seq
...............................................uuggacgagcgguuuguAgagug........................................  2      1  seq
...............................................uuggacgagcgguuuguggagug........................................  402    0  seq
...............................................uuggacgagcgguuuguggagAg........................................  1      1  seq
...............................................uuggacgagcgguuuguggagugU.......................................  35     1  seq
...............................................uuggacgagcgguuuguggagugG.......................................  1      1  seq
................................................uggacgagcgguuuguggagug........................................  7      0  seq
.................................................ggacgagcgguuuguggagugU.......................................  7      1  seq
..................................................gacgagcgguuuguggagug........................................  1      0  seq
....................................................................ugccagaucuaacucuucc.......................  1      0  seq
```

Star **Mature**

```
cuugauguucugccaggugcgaugaguuggaggauugggucucagcauuggacgagcgguuuguggagugccagaucuaacucuuccagcucaucccacucuggaccucc
....................................................................ugccagaucuaacucuuccag.....................   3      0   seq
....................................................................ugccagaucuaacucuuccagc....................   134    0   seq
....................................................................Ggccagaucuaacucuuccagc....................   1      1   seq
....................................................................ugccagaucuaacucuuccagU....................   2      1   seq
.....................................................................Uccagaucuaacucuuccagcu...................   1      1   seq
.....................................................................Cccagaucuaacucuuccagcu...................   128    1   seq
.....................................................................Cccagaucuaacucuuccagcuc..................   1      1   seq
.....................................................................Cccagaucuaacucuuccagcuca.................   225    1   seq
.....................................................................Accagaucuaacucuuccagcuca.................   11     1   seq
.....................................................................gccagaucuaacucuuccagcuca.................   1      0   seq
.....................................................................Accagaucuaacucuuccagcucau................   2      1   seq
......................................................................ccagaucuaacucuuccG......................   1      1   seq
......................................................................ccagaucuaacucuucca......................   36     0   seq
......................................................................ccagaucuaacucuuccU......................   81     1   seq
......................................................................ccagaucAaacucuuccag.....................   1      1   seq
......................................................................ccagaucuaacucuucUag.....................   1      1   seq
......................................................................cAagaucuaacucuuccag.....................   1      1   seq
......................................................................ccagaucuaacuAuuccag.....................   1      1   seq
......................................................................ccagUucuaacucuuccag.....................   1      1   seq
......................................................................ccagauAuaacucuuccag.....................   1      1   seq
......................................................................ccagaucuGacucuuccag.....................   1      1   seq
......................................................................ccagaucuaacucuuccaU.....................   19     1   seq
......................................................................ccagaucuaacucGuccag.....................   1      1   seq
......................................................................ccagaucuaacucuuccag.....................   1436   0   seq
......................................................................Ucagaucuaacucuuccag.....................   1      1   seq
......................................................................ccagaucuaacAcuuccag.....................   1      1   seq
......................................................................ccagaucuaUcucuuccagc....................   2      1   seq
......................................................................ccagaucuaacucuucUagc....................   13     1   seq
......................................................................ccagaucuaacucuCccagc....................   1      1   seq
......................................................................ccagaucuaacucuAccagc....................   2      1   seq
......................................................................ccagaucuaacucuucGagc....................   7      1   seq
......................................................................ccagaucuGacucuuccagc....................   3      1   seq
......................................................................Acagaucuaacucuuccagc....................   9      1   seq
......................................................................ccagaucuaacucCuccagc....................   3      1   seq
......................................................................ccagaucuaacucuuAcagc....................   9      1   seq
......................................................................ccagaAcuaacucuuccagc....................   7      1   seq
......................................................................ccagaucuaacucuuccGgc....................   4      1   seq
......................................................................ccagaucuaacucuuccagc....................   17986  0   seq
......................................................................ccagaCcuaacucuuccagc....................   2      1   seq
......................................................................ccagaucuaacucuuccUgc....................   2      1   seq
......................................................................ccagaucuaacucuuGcagc....................   1      1   seq
......................................................................ccagaucuaacucuuccagU....................   146    1   seq
......................................................................ccagaucuaacCcuuccagc....................   3      1   seq
......................................................................ccagaucuaacucAuccagc....................   3      1   seq
......................................................................ccagaucuaacucGuccagc....................   1      1   seq
......................................................................Gcagaucuaacucuuccagc....................   1      1   seq
......................................................................ccagaucuaacucuuccaUc....................   1      1   seq
......................................................................ccagaucuaGcucuuccagc....................   4      1   seq
......................................................................ccagaucAaacucuuccagc....................   7      1   seq
......................................................................ccagaucuaacuAuuccagc....................   5      1   seq
......................................................................ccagCucuaacucuuccagc....................   2      1   seq
......................................................................ccagGucuaacucuuccagc....................   3      1   seq
......................................................................Ucagaucuaacucuuccagc....................   10     1   seq
......................................................................ccaUaucuaacucuuccagc....................   1      1   seq
......................................................................ccagaucuaacucuuccaAc....................   2      1   seq
......................................................................ccagaucuaacuUuuccagc....................   1      1   seq
......................................................................ccagaucuaacucuuUcagc....................   1      1   seq
......................................................................cUagaucuaacucuuccagc....................   10     1   seq
......................................................................ccagaucuaaAucuuccagc....................   4      1   seq
......................................................................ccagaucuaacucuuccagG....................   1      1   seq
......................................................................ccNgaucuaacucuuccagc....................   1      1   seq
......................................................................ccagauGuaacucuuccagc....................   1      1   seq
......................................................................ccagaucuaacucuucAagc....................   3      1   seq
......................................................................ccUgaucuaacucuuccagc....................   3      1   seq
......................................................................ccagaucuaaGucuuccagc....................   2      1   seq
......................................................................ccagauUuaacucuuccagc....................   7      1   seq
......................................................................ccagaucuaacucuuccagA....................   473    1   seq
......................................................................ccagaNcuaacucuuccagc....................   1      1   seq
......................................................................cAagaucuaacucuuccagc....................   10     1   seq
......................................................................ccagaucuaacucuGccagc....................   3      1   seq
```

Star   Mature

```
cuugauguucugccaggugcgaugaguuggaggauugggucucagcauuggacgagcgguuuguggagugccagaucuaacucuuccagcucaucccacucuggaccucc
......................................................................ccagaucuaacAcuuccagc....................    6       1   seq
......................................................................ccagaucuaacucNuccagc....................    1       1   seq
......................................................................ccagaucCaacucuuccagc....................    5       1   seq
......................................................................ccagauAuaacucuuccagc....................    16      1   seq
......................................................................ccagUucuaacucuuccagc....................    1       1   seq
......................................................................cNagaucuaacucuuccagc....................    1       1   seq
......................................................................ccagaucuaacucuuccCgc....................    1       1   seq
......................................................................cGagaucuaacucuuccagc....................    2       1   seq
......................................................................ccagaucuaacuGuuccagc....................    1       1   seq
......................................................................ccaAaucuaacucuuccagc....................    1       1   seq
......................................................................ccagaucuaacucuuccagUu...................    40      1   seq
......................................................................cGagaucuaacucuuccagcu...................    7       1   seq
......................................................................ccagaucuaacucuuccaAcu...................    10      1   seq
......................................................................ccagCucuaacucuuccagcu...................    21      1   seq
......................................................................ccagaucuaacucuuGcagcu...................    10      1   seq
......................................................................ccagaucuaacucuCccagcu...................    15      1   seq
......................................................................ccagaAcuaacucuuccagcu...................    49      1   seq
......................................................................ccagaucCaacucuuccagcu...................    24      1   seq
......................................................................ccagaucuaacucuuccagAu...................    95      1   seq
......................................................................ccagauAuaacucuuccagcu...................    182     1   seq
......................................................................ccagaucuCacucuuccagcu...................    2       1   seq
......................................................................ccagaucuaacucuuAcagcu...................    32      1   seq
......................................................................ccagaucuaaNucuuccagcu...................    1       1   seq
......................................................................ccagaucuaacuUuuccagcu...................    7       1   seq
......................................................................ccagaucuaacucuuUcagcu...................    24      1   seq
......................................................................ccagaucuGacucuuccagcu...................    32      1   seq
......................................................................cAagaucuaacucuuccagcu...................    52      1   seq
......................................................................ccagaucuaacucGuccagcu...................    3       1   seq
......................................................................ccagaucuaacucuAccagcu...................    50      1   seq
......................................................................ccagaucuaacGcuuccagcu...................    2       1   seq
......................................................................ccagaucuaaAucuuccagcu...................    25      1   seq
......................................................................ccagaucuaCcucuuccagcu...................    1       1   seq
......................................................................ccagaGcuaacucuuccagcu...................    1       1   seq
......................................................................ccagaucuaGcucuuccagcu...................    27      1   seq
......................................................................ccagauUuaacucuuccagcu...................    32      1   seq
......................................................................cUagaucuaacucuuccagcu...................    126     1   seq
......................................................................Gcagaucuaacucuuccagcu...................    6       1   seq
......................................................................ccNgaucuaacucuuccagcu...................    1       1   seq
......................................................................Acagaucuaacucuuccagcu...................    81      1   seq
......................................................................ccagaucuaacucuucUagcu...................    177     1   seq
......................................................................ccagaucuaacucuuccCgcu...................    4       1   seq
......................................................................ccagUucuaacucuuccagcu...................    17      1   seq
......................................................................ccagaucuaacucuucGagcu...................    25      1   seq
......................................................................ccagaucuaacucuuccagcN...................    2       1   seq
......................................................................ccUgaucuaacucuuccagcu...................    7       1   seq
......................................................................ccagaucuaacucuuccagcG...................    643     1   seq
......................................................................ccaAaucuaacucuuccagcu...................    32      1   seq
......................................................................ccagaucuUacucuuccagcu...................    4       1   seq
......................................................................ccaUaucuaacucuuccagcu...................    9       1   seq
......................................................................ccagauGuaacucuuccagcu...................    4       1   seq
......................................................................ccagaucuaacucuuccGgcu...................    37      1   seq
......................................................................ccagaucuaacucAuccagcu...................    48      1   seq
......................................................................Ncagaucuaacucuuccagcu...................    46      1   seq
......................................................................ccagaucuaacucuuccagcC...................    21      1   seq
......................................................................ccagaucuaacucuuccaCcu...................    2       1   seq
......................................................................ccGgaucuaacucuuccagcu...................    32      1   seq
......................................................................ccagaucuaacuGuuccagcu...................    5       1   seq
......................................................................ccagaucuaacucuuccagcu...................    183222  0   seq
......................................................................ccagaucGaacucuuccagcu...................    4       1   seq
......................................................................cNagaucuaacucuuccagcu...................    3       1   seq
......................................................................ccagaucuaacucuucAagcu...................    69      1   seq
......................................................................ccagaucuaaGucuuccagcu...................    5       1   seq
......................................................................ccagaucuaacCcuuccagcu...................    15      1   seq
......................................................................ccagaucuNacucuuccagcu...................    2       1   seq
......................................................................ccagaucuaacucuuccagGu...................    5       1   seq
......................................................................ccagaucuaacucuuccagcA...................    84      1   seq
......................................................................ccagaucuaacucuGccagcu...................    7       1   seq
......................................................................ccagaucuaaUucuuccagcu...................    9       1   seq
......................................................................ccagaNcuaacucuuccagcu...................    6       1   seq
......................................................................ccagaucuaacucCuccagcu...................    16      1   seq
```

Star Mature

```
cuugauguucugccaggugcgaugaguuggaggauugggucucagcauuggacgagcgguuuguggagugccagaucuaacucuuccagcucaucccacucuggaccucc
......................................................................ccagaCcuaacucuuccagcu...................    10      1  seq
......................................................................ccagaucAaacucuuccagcu...................    76      1  seq
......................................................................ccagaucuaacAcuuccagcu...................    57      1  seq
......................................................................ccagaucuaacucuuccaUcu...................    11      1  seq
......................................................................ccaCaucuaacucuuccagcu...................    13      1  seq
......................................................................ccagaucuaUcucuuccagcu...................    23      1  seq
......................................................................ccCgaucuaacucuuccagcu...................    3       1  seq
......................................................................ccagGucuaacucuuccagcu...................    20      1  seq
......................................................................Ucagaucuaacucuuccagcu...................    175     1  seq
......................................................................ccagaucuaacuAuuccagcu...................    36      1  seq
......................................................................ccagaucuaacucuuccUgcu...................    6       1  seq
......................................................................ccagaucuaaUucuuccagcuc..................    27      1  seq
......................................................................ccagaucuaacucuuccagNuc..................    2       1  seq
......................................................................ccagaNcuaacucuuccagcuc..................    14      1  seq
......................................................................ccagaucuaacucuuccagcuc..................    356655  0  seq
......................................................................Ucagaucuaacucuuccagcuc..................    244     1  seq
......................................................................ccagaucuaacucuuccaCcuc..................    13      1  seq
......................................................................cNagaucuaacucuuccagcuc..................    9       1  seq
......................................................................ccagaucuaacucuuGcagcuc..................    16      1  seq
......................................................................ccagaAcuaacucuuccagcuc..................    134     1  seq
......................................................................ccagauAuaacucuuccagcuc..................    359     1  seq
......................................................................ccagaucuaacucGuccagcuc..................    8       1  seq
......................................................................ccagaucuaacucuuccUgcuc..................    16      1  seq
......................................................................ccagaucuaacucuAccagcuc..................    121     1  seq
......................................................................ccagGucuaacucuuccagcuc..................    41      1  seq
......................................................................ccagaCcuaacucuuccagcuc..................    56      1  seq
......................................................................ccagaucuaacucuuccagcNc..................    2       1  seq
......................................................................Acagaucuaacucuuccagcuc..................    130     1  seq
......................................................................ccagaucuaCcucuuccagcuc..................    3       1  seq
......................................................................ccagaucuaacucNuccagcuc..................    1       1  seq
......................................................................ccagaucuaacucuucGagcuc..................    34      1  seq
......................................................................ccagauUuaacucuuccagcuc..................    51      1  seq
......................................................................ccagaucuaUcucuuccagcuc..................    46      1  seq
......................................................................cAagaucuaacucuuccagcuc..................    116     1  seq
......................................................................ccagaucuaacuUuuccagcuc..................    14      1  seq
......................................................................ccagaucuaacucuuNcagcuc..................    1       1  seq
......................................................................ccagaucuaacucuucNagcuc..................    1       1  seq
......................................................................ccagaucuGacucuuccagcuc..................    65      1  seq
......................................................................ccagaucuaacucCuccagcuc..................    53      1  seq
......................................................................ccagaucuaacucuucAagcuc..................    108     1  seq
......................................................................ccagaucAaacucuuccagcuc..................    154     1  seq
......................................................................ccaUaucuaacucuuccagcuc..................    17      1  seq
......................................................................ccCgaucuaacucuuccagcuc..................    6       1  seq
......................................................................ccagauGuaacucuuccagcuc..................    17      1  seq
......................................................................ccagaucuaacucuuccaAcuc..................    19      1  seq
......................................................................ccagUucuaacucuuccagcuc..................    31      1  seq
......................................................................ccagaucuaacucuuccaNcuc..................    1       1  seq
......................................................................ccagaucuaacucuuccGgcuc..................    86      1  seq
......................................................................ccagaucuaaAucuuccagcuc..................    48      1  seq
......................................................................ccagaGcuaacucuuccagcuc..................    5       1  seq
......................................................................ccagaucuaacucuuccagGuc..................    5       1  seq
......................................................................ccagaucuaacucuCccagcuc..................    57      1  seq
......................................................................ccUgaucuaacucuuccagcuc..................    28      1  seq
......................................................................ccagaucuaNcucuuccagcuc..................    3       1  seq
......................................................................cGagaucuaacucuuccagcuc..................    27      1  seq
......................................................................ccagaucuaacucuuccagcuG..................    26      1  seq
......................................................................ccagaucuaacucuuccagUuc..................    22      1  seq
......................................................................ccaAaucuaacucuuccagcuc..................    79      1  seq
......................................................................ccagaucuaacucuuccaUcuc..................    23      1  seq
......................................................................ccagaucuaacucuucUagcuc..................    344     1  seq
......................................................................ccGgaucuaacucuuccagcuc..................    76      1  seq
......................................................................ccagaucuaacGcuuccagcuc..................    7       1  seq
......................................................................ccagaucuaGcucuuccagcuc..................    42      1  seq
......................................................................ccagaucuaacucuuUcagcuc..................    49      1  seq
......................................................................ccagaucuUacucuuccagcuc..................    4       1  seq
......................................................................ccagaucuaacucuuccagcuA..................    250     1  seq
......................................................................ccagaucuaaNucuuccagcuc..................    2       1  seq
......................................................................Gcagaucuaacucuuccagcuc..................    115     1  seq
......................................................................ccagaucuaacucuGccagcuc..................    17      1  seq
......................................................................ccagaucuaacucAuccagcuc..................    76      1  seq
```

Star Mature

```
cuugauguucugccaggugcgaugaguuggaggauugggucucagcauuggacgagcgguuuguggagugccagaucuaacucuuccagcucaucccacucuggaccucc
......................................................................ccagaucuaacCcuuccagcuc..................    30      1       seq
......................................................................cUagaucuaacucuuccagcuc..................    238     1       seq
......................................................................ccagaucuaacucuuccagcGc..................    4       1       seq
......................................................................ccagaucuaaGucuuccagcuc..................    5       1       seq
......................................................................ccagaucuaacucuuccagcCc..................    40      1       seq
......................................................................Ncagaucuaacucuuccagcuc..................    59      1       seq
......................................................................ccagaucuaacucuuccagcAc..................    107     1       seq
......................................................................ccagaucuaacAcuuccagcuc..................    125     1       seq
......................................................................ccagaucGaacucuuccagcuc..................    3       1       seq
......................................................................ccagaucuaacucuuccagAuc..................    165     1       seq
......................................................................ccagaucuaacucuuccCgcuc..................    13      1       seq
......................................................................ccagCucuaacucuuccagcuc..................    44      1       seq
......................................................................ccagaucCaacucuuccagcuc..................    48      1       seq
......................................................................ccagaucuaacucuuccagcuN..................    1       1       seq
......................................................................ccagaucuaacucuuccagcuU..................    2247    1       seq
......................................................................ccaCaucuaacucuuccagcuc..................    24      1       seq
......................................................................ccagaucuaacuGuuccagcuc..................    13      1       seq
......................................................................ccagaucuaacucuuAcagcuc..................    88      1       seq
......................................................................ccagaucuCacucuuccagcuc..................    5       1       seq
......................................................................ccagaucuaacuAuuccagcuc..................    105     1       seq
......................................................................ccagauAuaacucuuccagcuca.................    650     1       seq
......................................................................ccagCucuaacucuuccagcuca.................    81      1       seq
......................................................................ccagaucuaacucuCccagcuca.................    156     1       seq
......................................................................ccUgaucuaacucuuccagcuca.................    41      1       seq
......................................................................ccagaucuaacucuuUcagcuca.................    97      1       seq
......................................................................Gcagaucuaacucuuccagcuca.................    34      1       seq
......................................................................ccagaucuaaAucuuccagcuca.................    111     1       seq
......................................................................ccagaucuaacucuuccagcucG.................    542     1       seq
......................................................................ccagaucuaGcucuuccagcuca.................    111     1       seq
......................................................................ccagaGcuaacucuuccagcuca.................    13      1       seq
......................................................................ccagauGuaacucuuccagcuca.................    31      1       seq
......................................................................ccagaucuaacucCuccagcuca.................    202     1       seq
......................................................................ccagaucuaacucuuccagcAca.................    248     1       seq
......................................................................ccagaucuaacuAuuccagcuca.................    227     1       seq
......................................................................ccagaucuaaGucuuccagcuca.................    20      1       seq
......................................................................ccagaucuaacGcuuccagcuca.................    8       1       seq
......................................................................ccagaucuaacucuuccaUcuca.................    59      1       seq
......................................................................ccagaNcuaacucuuccagcuca.................    21      1       seq
......................................................................ccagaucuaacucGuccagcuca.................    24      1       seq
......................................................................ccagaucuaCcucuuccagcuca.................    5       1       seq
......................................................................ccagaucuaacucuuGcagcuca.................    46      1       seq
......................................................................ccagaucuaacucuuccagNuca.................    1       1       seq
......................................................................ccagaucAaacucuuccagcuca.................    368     1       seq
......................................................................ccagaucuaacucuuccagcGca.................    31      1       seq
......................................................................ccagaucuaacucuuccagcuAa.................    495     1       seq
......................................................................cAagaucuaacucuuccagcuca.................    258     1       seq
......................................................................ccagaucuaacucuuccagcuUa.................    476     1       seq
......................................................................ccagaucuaacucuucGagcuca.................    114     1       seq
......................................................................cGagaucuaacucuuccagcuca.................    48      1       seq
......................................................................ccagaCcuaacucuuccagcuca.................    96      1       seq
......................................................................ccagUucuaacucuuccagcuca.................    94      1       seq
......................................................................ccagaucuaaUucuuccagcuca.................    64      1       seq
......................................................................ccagaucuaacucuAccagcuca.................    309     1       seq
......................................................................ccGgaucuaacucuuccagcuca.................    217     1       seq
......................................................................ccCgaucuaacucuuccagcuca.................    16      1       seq
......................................................................ccagaucuUacucuuccagcuca.................    10      1       seq
......................................................................ccagaAcuaacucuuccagcuca.................    253     1       seq
......................................................................ccagGucuaacucuuccagcuca.................    85      1       seq
......................................................................ccagaucuaacuGuuccagcuca.................    20      1       seq
......................................................................ccagaucuaacucAuccagcuca.................    188     1       seq
......................................................................ccagaucuaacucuuccagcCca.................    173     1       seq
......................................................................Ncagaucuaacucuuccagcuca.................    218     1       seq
......................................................................ccagaucuaacucuuccagAuca.................    378     1       seq
......................................................................ccagaucuaacucuuccagcuNa.................    3       1       seq
......................................................................ccagauUuaacucuuccagcuca.................    151     1       seq
......................................................................ccagaucuaacucuuccagcNca.................    5       1       seq
......................................................................Acagaucuaacucuuccagcuca.................    336     1       seq
......................................................................ccagaucuaacuUuuccagcuca.................    53      1       seq
......................................................................ccagaucuaacucuNccagcuca.................    1       1       seq
......................................................................ccagaucuaacucuuccaCcuca.................    32      1       seq
```

```
                       Star                                        Mature
cuugauguucugccaggugcgaugaguuggaggauugggucucagcauuggacgagcgguuuguggagugccagaucuaacucuuccagcucaucccacucuggaccucc
......................................................................ccagaucuaacucuuccagcucC.................  388     1   seq
......................................................................Ucagaucuaacucuuccagcuca.................  598     1   seq
......................................................................ccagaucuaacucuuccagcuGa.................  44      1   seq
......................................................................ccagaucuaacucuucNagcuca.................  1       1   seq
......................................................................cNagaucuaacucuuccagcuca.................  22      1   seq
......................................................................ccaUaucuaacucuuccagcuca.................  69      1   seq
......................................................................ccagaucuaacAcuuccagcuca.................  290     1   seq
......................................................................ccagaucuaacucuuccagGuca.................  22      1   seq
......................................................................ccagaucuaUcucuuccagcuca.................  82      1   seq
......................................................................ccaAaucuaacucuuccagcuca.................  144     1   seq
......................................................................ccagaucuaacucuGccagcuca.................  33      1   seq
......................................................................ccaCaucuaacucuuccagcuca.................  43      1   seq
......................................................................ccagaucuaNcucuuccagcuca.................  5       1   seq
......................................................................ccagaucuaacucuuccagUuca.................  51      1   seq
......................................................................ccagaucuaacucuuccaAcuca.................  53      1   seq
......................................................................ccagaucuCacucuuccagcuca.................  13      1   seq
......................................................................ccagaucuaacucuuAcagcuca.................  224     1   seq
......................................................................cUagaucuaacucuuccagcuca.................  617     1   seq
......................................................................ccagaucuaacucuucAagcuca.................  265     1   seq
......................................................................ccagaucuaacucuuccCgcuca.................  28      1   seq
......................................................................ccagaucuGacucuuccagcuca.................  171     1   seq
......................................................................ccagaucuaacucuuccUgcuca.................  29      1   seq
......................................................................ccagaucuaacucuucUagcuca.................  828     1   seq
......................................................................ccagaucuaacCcuuccagcuca.................  79      1   seq
......................................................................ccagaucuaacucuuccGgcuca.................  172     1   seq
......................................................................ccagaucuaacucuuccaNcuca.................  1       1   seq
......................................................................ccagaucuaacucuuccagcucN.................  24      1   seq
......................................................................ccagaucuaacucuuccagcucU.................  45192   1   seq
......................................................................ccagaucCaacucuuccagcuca.................  97      1   seq
......................................................................ccagaucGaacucuuccagcuca.................  19      1   seq
......................................................................ccNgaucuaacucuuccagcuca.................  3       1   seq
......................................................................ccagaucuaacucuuccagcuca.................  838706  0   seq
......................................................................ccUgaucuaacucuuccagcucau................  1       1   seq
......................................................................ccagaucuaacucuuccagcucCu................  82      1   seq
......................................................................ccagaucuaacucuuccagcCcau................  1       1   seq
......................................................................ccagaucuaacucuuccagcucGu................  1       1   seq
......................................................................ccagaucuaacucGuccagcucau................  1       1   seq
......................................................................ccagaucuaacucuuccagAucau................  12      1   seq
......................................................................ccagaucuaacucuuccagcucaG................  38      1   seq
......................................................................ccGgaucuaacucuuccagcucau................  2       1   seq
......................................................................ccagaucuaacucuucUagcucau................  17      1   seq
......................................................................ccagaucuaacucuuccagcAcau................  5       1   seq
......................................................................ccagaucuaacucuCccagcucau................  1       1   seq
......................................................................ccagaucuaacuUuuccagcucau................  2       1   seq
......................................................................ccagaucuaacucuuccGgcucau................  3       1   seq
......................................................................ccagaucuaacucuuccaUcucau................  1       1   seq
......................................................................cAagaucuaacucuuccagcucau................  7       1   seq
......................................................................ccagaucuaUcucuuccagcucau................  2       1   seq
......................................................................ccagaucAaacucuuccagcucau................  4       1   seq
......................................................................ccagaucuaacucuuUcagcucau................  3       1   seq
......................................................................ccagaucuaacucuuccaAcucau................  1       1   seq
......................................................................ccagaucuaacuAuuccagcucau................  5       1   seq
......................................................................ccagaucuaacucuuccagcucau................  15801   0   seq
......................................................................ccagaucuaacucuAccagcucau................  5       1   seq
......................................................................ccagaucuaacucuuccagcucaA................  2133    1   seq
......................................................................ccagaucuaacucuuccagcGcau................  1       1   seq
......................................................................ccagaucuaacucuuccagcucUu................  192     1   seq
......................................................................ccagaucuaacucuucNagcucau................  1       1   seq
......................................................................ccagaucuaacAcuuccagcucau................  6       1   seq
......................................................................ccagaucuaacuGuuccagcucau................  1       1   seq
......................................................................ccagaucuaacucuuAcagcucau................  4       1   seq
......................................................................ccagaucuaacucuuccagcucaN................  1       1   seq
......................................................................ccagaucuaaAucuuccagcucau................  3       1   seq
......................................................................ccagaucuaaUucuuccagcucau................  1       1   seq
......................................................................Ncagaucuaacucuuccagcucau................  3       1   seq
......................................................................ccagaucuaacucuuccagcuGau................  2       1   seq
......................................................................ccagaucuGacucuuccagcucau................  5       1   seq
......................................................................ccagaAcuaacucuuccagcucau................  3       1   seq
......................................................................ccagauGuaacucuuccagcucau................  1       1   seq
......................................................................ccagaucuaaGucuuccagcucau................  1       1   seq
```

Star **Mature**

cuugauguucugccaggugcgaugaguuggaggauugggucucagcauuggacgagcgguuuguggagugccagaucuaacucuuccagcucaucccacucuggaccucc			
....ccagaucuaacucuucAagcucau................	5	1	seq
....ccagauUuaacucuuccagcucau................	2	1	seq
....ccagaucuaacucCuccagcucau................	2	1	seq
....ccagaucuaacucAuccagcucau................	1	1	seq
....ccagaucuaacucuuccagcucaC................	923	1	seq
....ccagauAuaacucuuccagcucau................	11	1	seq
....ccagaGcuaacucuuccagcucau................	2	1	seq
....ccagaucuaacucuuccagcuUau................	8	1	seq
....ccagaucuaacucuuccagcuAau................	11	1	seq
....ccagaucuaacucuucGagcucau................	5	1	seq
....ccagaucGaacucuuccagcucau................	1	1	seq
....ccagaucuaacucuuccaCcucau................	2	1	seq
....ccagGucuaacucuuccagcucau................	4	1	seq
....Acagaucuaacucuuccagcucau................	6	1	seq
....ccagCucuaacucuuccagcucau................	3	1	seq
....Ucagaucuaacucuuccagcucau................	10	1	seq
....cUagaucuaacucuuccagcucau................	17	1	seq
....ccagaucuaacucuuccagcucaCc...............	2	1	seq
....ccagaucuaacucuuccagcucauU...............	11428	1	seq
....ccagaucuaacucuuccagcucauA...............	661	1	seq
....ccagaucuaacucuuccagcucauG...............	7	1	seq
....ccagaucuaacCcuuccagcucauc...............	1	1	seq
....ccagaucuaacucuuccagcucauc...............	76	0	seq
....ccagaucuaacucuuccagcuAauc...............	1	1	seq
....ccagaucuaacucuuccagcucUuc...............	8	1	seq
....ccagauAuaacucuuccagcucauc...............	1	1	seq
....ccagaucuaacucuuccagcucaAc...............	2	1	seq
....ccagaucuaacucuuccagcucaucU..............	141	1	seq
....cagaucuaacucuuccag.....................	1	0	seq
....cagaucuaacucuuccagc....................	36	0	seq
....cagaucuaacuAuuccagcu...................	5	1	seq
....cagaucuaacucuCccagcu...................	1	1	seq
....cagaucuaacucuuAcagcu...................	2	1	seq
....cagaucAaacucuuccagcu...................	2	1	seq
....Uagaucuaacucuuccagcu...................	2	1	seq
....cagauAuaacucuuccagcu...................	7	1	seq
....cagaucuaacucuuccagcG...................	2	1	seq
....cagaucuaacucuAccagcu...................	1	1	seq
....cagaucuaacAcuuccagcu...................	1	1	seq
....cagaucuaacucuuccagcu...................	1058	0	seq
....cUgaucuaacucuuccagcu...................	1	1	seq
....cagaAcuaacucuuccagcu...................	1	1	seq
....cCgaucuaacucuuccagcu...................	1	1	seq
....cagaucuaacucuuccagcA...................	1	1	seq
....cagaucuaacCcuuccagcu...................	1	1	seq
....cagaucuaacuAuuccagcuc..................	7	1	seq
....cagaucAaacucuuccagcuc..................	1	1	seq
....cagaucuaacucuuccagcAc..................	1	1	seq
....cagaucuaacucuuccagcuA..................	2	1	seq
....cagaucuaacucuuAcagcuc..................	5	1	seq
....cagaucuaacucuuccagcuc..................	1040	0	seq
....caUaucuaacucuuccagcuc..................	1	1	seq
....cagaucuaacucuuccagcuU..................	33	1	seq
....cagauAuaacucuuccagcuc..................	7	1	seq
....cagaucuaacucuAccagcuc..................	1	1	seq
....Nagaucuaacucuuccagcuc..................	1	1	seq
....Uagaucuaacucuuccagcuc..................	1	1	seq
....cagaucuaacucuuUcagcuc..................	1	1	seq
....cCgaucuaacucuuccagcuc..................	1	1	seq
....cagaucuaaAucuuccagcuc..................	1	1	seq
....cagaucuaGcucuuccagcuc..................	1	1	seq
....cagaucuaCcucuuccagcuca.................	1	1	seq
....cagaucuaacGcuuccagcuca.................	1	1	seq
....cagaucuGacucuuccagcuca.................	1	1	seq
....cagaucuaGcucuuccagcuca.................	1	1	seq
....cagaucuaacucCuccagcuca.................	1	1	seq
....cagaucuaacucuuccagcucC.................	1	1	seq
....caAaucuaacucuuccagcuca.................	1	1	seq
....cagaucuaaNucuuccagcuca.................	1	1	seq
....cagGucuaacucuuccagcuca.................	2	1	seq

Star **Mature**

```
cuugauguucugccaggugcgaugaguuggaggauugggucucagcauuggacgagcgguuuguggagugccagaucuaacucuuccagcucaucccacucuggaccucc
.......................................................................cagaucuaacucuuAcagcuca.................   19     1   seq
.......................................................................cagaucuaacucuuccaCcuca.................   1      1   seq
.......................................................................cagaucCaacucuuccagcuca.................   2      1   seq
.......................................................................cagaucuaacucuuccagcAca.................   1      1   seq
.......................................................................cCgaucuaacucuuccagcuca.................   3      1   seq
.......................................................................cagaCcuaacucuuccagcuca.................   1      1   seq
.......................................................................cagaucuaacucAuccagcuca.................   8      1   seq
.......................................................................cagaucuaacAcuuccagcuca.................   5      1   seq
.......................................................................cagauAuaacucuuccagcuca.................   14     1   seq
.......................................................................cagaucuaacucuuccagcucG.................   4      1   seq
.......................................................................cUgaucuaacucuuccagcuca.................   1      1   seq
.......................................................................cagaucuaacucuuccagAuca.................   1      1   seq
.......................................................................cagaucuaacucuuccagUuca.................   1      1   seq
.......................................................................cagaucuaacucuuccagcCca.................   1      1   seq
.......................................................................cagaucuaacuAuuccagcuca.................   11     1   seq
.......................................................................cagaucuaacucuucAagcuca.................   1      1   seq
.......................................................................Uagaucuaacucuuccagcuca.................   1      1   seq
.......................................................................cagaucAaacucuuccagcuca.................   6      1   seq
.......................................................................cagaucGaacucuuccagcuca.................   1      1   seq
.......................................................................cagauUuaacucuuccagcuca.................   1      1   seq
.......................................................................cagaucuaacucuucUagcuca.................   3      1   seq
.......................................................................cagaucuaacucuuccagcuca.................   4903   0   seq
.......................................................................cagaucuaacucuuccagcuUa.................   4      1   seq
.......................................................................Aagaucuaacucuuccagcuca.................   2      1   seq
.......................................................................cagaucuaacucuuccagcuAa.................   8      1   seq
.......................................................................cagaucuaacucGuccagcuca.................   1      1   seq
.......................................................................cagaucuaacucuuccCgcuca.................   2      1   seq
.......................................................................cagaucuaacucuAccagcuca.................   2      1   seq
.......................................................................cagaucuaacucuCccagcuca.................   1      1   seq
.......................................................................Nagaucuaacucuuccagcuca.................   1      1   seq
.......................................................................cagaucuaaAucuuccagcuca.................   4      1   seq
.......................................................................cagaucuaacucuuccagcucU.................   217    1   seq
.......................................................................cagaucuaacucuuccagcucau................   117    0   seq
.......................................................................cagaucuaacucuuccagcucaA................   13     1   seq
.......................................................................cagaucuaacucuuccagcucauU...............   23     1   seq
........................................................................agaucuaacucuuccagc....................   2      0   seq
........................................................................Cgaucuaacucuuccagcu...................   2      1   seq
........................................................................agaucuaacucuuccagcu...................   68     0   seq
........................................................................agaucuaacucuuccagcG...................   1      1   seq
........................................................................Cgaucuaacucuuccagcuc..................   1      1   seq
........................................................................agaucuaacucAuccagcuc..................   1      1   seq
........................................................................agaucuaacucuuAcagcuc..................   1      1   seq
........................................................................agaucuaacucuuccagcuc..................   141    0   seq
........................................................................agaucuaacucuuccagcucU.................   1      1   seq
........................................................................agaucuaacucuuccagcuUa.................   1      1   seq
........................................................................Cgaucuaacucuuccagcuca.................   3      1   seq
........................................................................agauUuaacucuuccagcuca.................   1      1   seq
........................................................................agaucuaacucuuccagcuca.................   173    0   seq
........................................................................agaucuaacucuuccagcucC.................   1      1   seq
........................................................................agaucuaacucuuccagcuAa.................   1      1   seq
........................................................................Ggaucuaacucuuccagcucau................   1      1   seq
........................................................................agaucuaacucuuccagcucaA................   8      1   seq
........................................................................agaucuaacucuuccagcucau................   23     0   seq
.........................................................................gaucuaacucuuccagcu...................   31     0   seq
.........................................................................Aaucuaacucuuccagcuc..................   1      1   seq
.........................................................................gaucuaacucuuccagcuc..................   185    0   seq
.........................................................................Uaucuaacucuuccagcuc..................   1      1   seq
.........................................................................gaucuaacucuucAagcuc..................   1      1   seq
.........................................................................gaucuaacucuuccagcuca.................   3      0   seq
..........................................................................aucuaacucuuccagcuc..................   3      0   seq
..........................................................................aucuaacucuuccagcuca.................   3      0   seq
...........................................................................Gcuaacucuuccagcuca.................   1      1   seq
...........................................................................Acuaacucuuccagcuca.................   1      1   seq
...........................................................................ucuaacucuuccagcuca.................   45     0   seq
```
